# Association between sleep duration and chest pain in US adults: A cross-sectional study

**DOI:** 10.3389/fpubh.2022.952075

**Published:** 2022-08-19

**Authors:** Wei Chen, Ji-ping Wang, Zi-min Wang, Peng-Cheng Hu, Yu Chen

**Affiliations:** ^1^Department of Thoracic Surgery, Taizhou First People's Hospital, Taizhou, China; ^2^Department of Thoracic Surgery, Taizhou Hospital of Zhejiang Province Affiliated to Wenzhou Medical University, Linhai, China; ^3^Department of Ophthalmology, The Second Affiliated Hospital of Chongqing Medical University, Chongqing Medical University, Chongqing, China

**Keywords:** sleep duration, chest pain, adults in US, cross-sectional study, U-shape

## Abstract

**Objective:**

Herein, we purposed to explore the association of sleep duration with chest pain among adults in US.

**Methods:**

This research work enrolled 13,274 subjects in the National Health and Nutrition Examination Survey (NHANES) from 2011 to 2018. The association of sleep duration with chest pain among adults in US was evaluated by Multivariable logistic regression.

**Results:**

To elucidate the association, we made adjustments for gender, BMI, diabetes, smoking status, drinking status, race, marital status, annual family income, hyperlipoidemia, Hypertension. Chest pain incidence decreased by 5% [OR = 0.95 (0.93, 0.98), *p* = 0.0004] for an increase in sleep duration by 1 h. A generalized additive model (GAM) was used to reseal a U-shaped relationship of sleep duration with incident chest pain. When duration of sleep was <6.5 h, chest pain incidence negatively correlated to sleep duration [OR = 0.77 (0.72, 0.82) *P* < 0.0001]. However, when sleep duration was ≥6.5 h, chest pain incidence rose with escalating sleep duration [OR = 1.07 (1.03, 1.11) *p* = 0.0014].

**Conclusions:**

Duration of sleep was established to be independently linked with an increase in the occurrence of chest pain. Excessive sleep, as much as insufficient sleep, increases the risk of chest pain. Both excessive sleep and insufficient sleep are associated with an increased risk of chest pain.

## Introduction

Chest pain is among the most frequently seen primary complaints in the emergency department (ED), with its incidence ranging between 5 and 12% (1, 2), and there are a wide range of its prospective causes, from benign to potentially life-threatening. It resulted in more than eight million visits yearly in the US and most subjects with acute chest pain are hospitalized for further assessment.

Sleep is an important health risk factor and plays a crucial role in a person's emotional and physical wellbeing (3, 4). Generally, optimal sleep duration is around 7 h in relation to subjective wellbeing and mental health (5, 6). Interestingly, only half of US adults report a habitual sleep time of 7 h or less (7).

Poor habits of sleeping are well recognized to cause health problems such as cardiovascular disease, cancer mortality, and mental health issues (5). However, few investigations have addressed the association of duration of sleep with chest pain and it still have been challenging. Hence, we carried out research to elucidate the association between duration of sleep and chest pain, to minimize incidences of chest pain to a certain extent. The study individuals come from the National Health and Nutrition Examination Survey (NHANES) during 2011–2018.

## Methods and materials

### Patient enrolment

**Figure 1:**
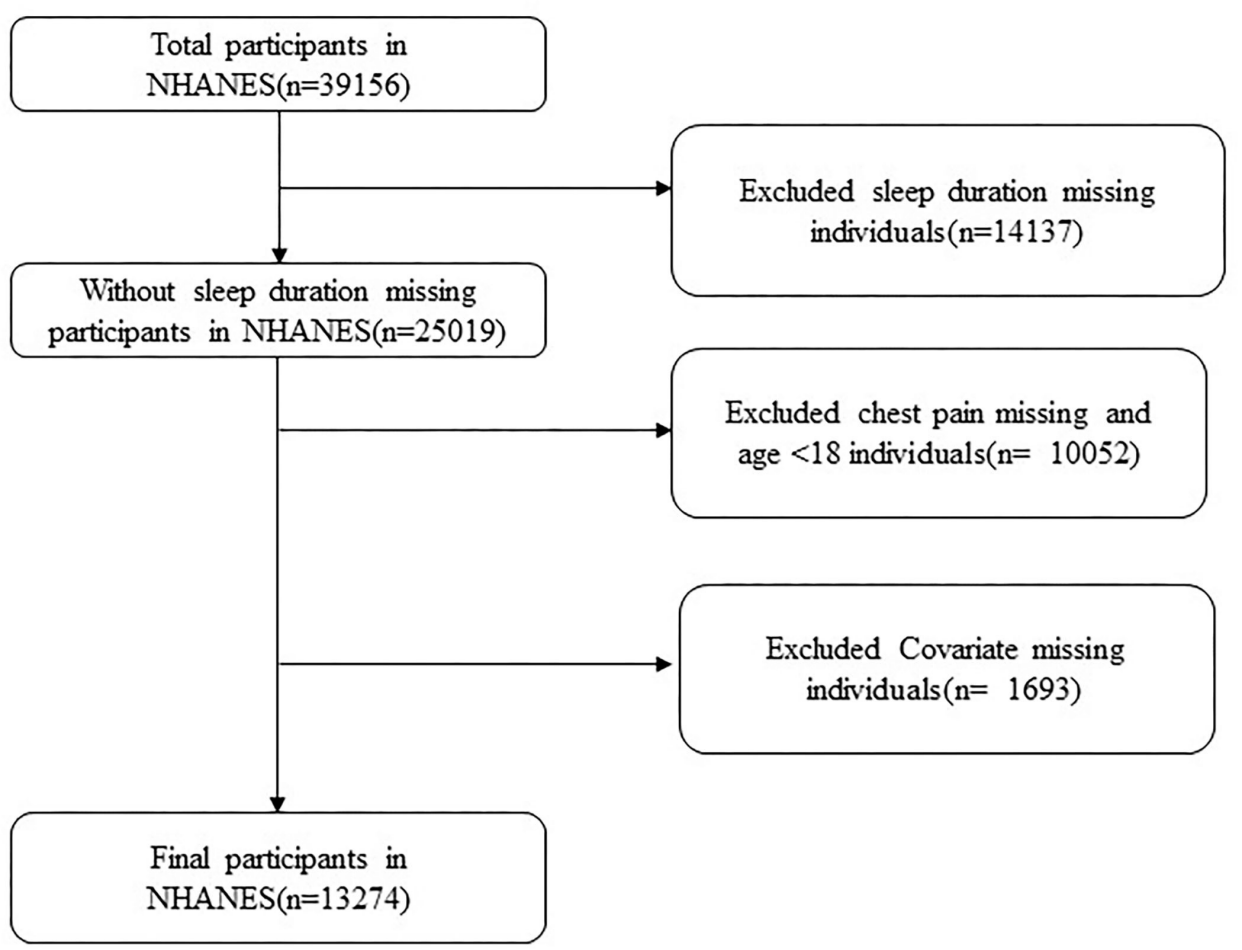
Flow chart of patient disposition.

The National Center for Health Statistics ethical review board authorized the procedures for the National Health and Nutrition Examination Survey (NHANES), and subjects gave their signed consent. The National Center for Health Statistics (NCHS) conducts representative cross-sectional surveys (NCHS). As the present study relied on existing NHANES data and did not generate any new data, no other local ethical approval is needed. We utilized data from NHANES III 2011–2018 of adult subjects (aged 18 and above). Criteria for exclusion consisted of subjects without chest pain or those lacking information of duration of sleep and covariates. Finally, 13,274 subjects were enrolled (Figure 1).

## Measurement

### Outcome ascertainment

The outcome of chest pain was explored with the Rose questionnaire (8). A trained interviewer conducted these questions during a computer-facilitated personal interview (CAPI) at home. To minimize data entering mistakes, the CAPI system is developed with checks involving built-in consistency. CAPI additionally employs online assistance screens to aid interviewers define major phrases in the questionnaire. Chest pain was created from a question “Have you ever had any pain or discomfort in your chest?”. Therefore, subjects were stratified into two groups: pain and no pain.

### Exposure measurement

Participants reported the duration of their sleep during a normal weekday or workday by self-reporting. During 2011–2014, duration of sleep was determined using a question asked of NHANES subjects: “How much sleep do you get (h)?”. During the 2014–2018 cycle, duration of sleep was generated from a question asked to NHANES participants: “How much sleep do you usually get at night on weekdays or workdays.” We categorized the duration of sleep as <6 h (short), 6 h ≤ sleep duration <8 h (midrange), as well as ≥8 h (long).

### Covariate assessment

Covariate selection was on the basis of previous investigations and clinical experience. the following variables was included: gender, age, BMI, diabetes, race, marital status, annual family income, hyperlipoidemia, Hypertension.

## Statistical analysis

Data are shown as mean ± SD or median (interquartile) for continuous variables. When missing values consisted of continuous variables, they were complemented with the median or mean. Comparison of variables among groups was performed with the use of the Student's *t*-test or Mann–Whitney *U*-test depending on the normality of the distribution, while the Fisher's Exact test was used to compare categorical variables. The frequency or percentage value for categorical variables is shown on the graph. Logistic regression models were adopted to explore the relationship of duration of sleep with chest pain. We adopted unadjusted along with multivariate adjusted models. the following variables was included: age and gender, BMI, diabetes, smoking status, drinking status, race, marital status, annual family income, hyperlipoidemia, Hypertension. Trend tests were performed using linear regression, with duration of sleep divided into three groups (<6 h (short), 6 h ≤ sleep duration <8 h (midrange), ≥8 h (long) as a continuous variable in the models.

To determine the non-linear relationship between duration of sleep and chest pain, a generalized additive model (GAM) was used. A two-piecewise linear regression approach was adopted to explore the threshold effect of sleep duration on chest pain. The inflection point was computed automatically using the recursive approach if the chest pain and sleep duration were obvious in a smoothed curve.

Data was analyzed using R 3.3.2 (http://www.R-project.org), along with EmpowerStats (X&Y Solutions, Boston, MA). P < 0.05 indicates statistical significance.

## Results

This research work enrolled 13,274 subjects (mean age: 59.72 ± 12.18 years, male: 48.17%) at baseline assessment, Chest pain participants were more inclined to have hypertension, a low annual family income, hyperlipoidemia, be diabetic. Chest pain is more common in participants with short sleep duration. In Table 1, the participant's characteristics are summarized in more detail.

**Table 1:** Baseline characteristics of the study participants.

**Characteristics**	**Total (*n* = 13274)**	**No pain (*n* = 9828)**	**Pain (*n* = 3446)**	***p***
Gender (%)				0.343
Male	6,394 (48.17)	4,758 (48.41)	1,636 (47.48)	
Female	6,880 (51.83)	5,070 (51.59)	1,810 (52.52)	
Age (Mean+SD)	59.72 ± 12.18	59.44 ± 12.23	60.50 ± 12.00	<0.001
BMI (Mean+SD)	29.57 ± 6.89	29.18 ± 6.64	30.68 ± 7.43	
Race *n* (%)				<0.001
Mexican American	1,610 (12.13)	1,230 (12.52)	380 (11.03)	
Other hispanic	1,375 (10.36)	1,014 (10.32)	361 (10.48)	
Non-Hispanic White	5,264 (39.66)	3,722 (37.87)	1,542 (44.75)	
Non-Hispanic Black	3,020 (22.75)	2,237 (22.76)	783 (22.72)	
Non-Hispanic Asian	1,604 (12.08)	1,392 (14.16)	212 (6.15)	
Other race	401 (3.02)	233 (2.37)	168 (4.88)	
Marrage *n* (%)				<0.001
Married	7,504 (56.53)	5,776 (58.77)	1,728 (50.15)	
Widowed	1,546 (11.65)	1,093 (11.12)	453 (13.15)	
Divorced	1,947 (14.67)	1,359 (13.83)	588 (17.06)	
Separated	505 (3.80)	331 (3.37)	174 (5.05)	
Never married	1,156 (8.71)	817 (8.31)	339 (9.84)	
Living with partner	616 (4.64)	452 (4.60)	164 (4.76)	
Annual family income				<0.001
Under $20,000	2,835(21.36)	1,867(18.99)	968(28.09)	
$20,000 and Over	10,439(78.64)	7,961(81.00)	2,478(77.91)	
Hypertension *n* (%)				<0.001
Yes	64,20 (48.37)	4,320 (43.96)	2,100 (60.94)	
No	6,854 (51.63)	5,508 (56.04)	1,346 (39.06)	
Hyperlipoidemia *n* (%)				<0.001
Yes	6,045 (45.54)	4,117 (41.89)	1,928 (55.95)	
No	7,229 (54.46)	5,711 (58.11)	1,518 (44.05)	
Diabetes *n* (%)				<0.001
Yes	2,659 (20.03)	1,785 (18.16)	874 (25.36)	
No	10,615 (79.97)	8,043 (81.84)	2,572 (74.67)	
Sleep duration (h)				<0.001
<6	1,641 (12.36)	1,069 (10.88)	572 (16.60)	
≥6, <8	6,023 (45.37)	4,587 (46.67)	1,436 (41.67)	
≧8	5,610 (42.26)	4,172 (42.45)	1,438 (41.73)	

Table 2 shows the relationship of sleep duration with chest pain. There is a difference in the odds ratios (95% confidence intervals) for chest pain for mid-range and long sleep durations compared to short sleep durations. In the crude model, the OR (95% CI) for chest pain association with sleep duration was 0.96(0.94, 0.99), the OR (95% CI) following age, as well as gender adjustments was 0.95 (0.93, 0.98), and OR (95% CI) following adjustment of age and gender, BMI, diabetes, race, marital status, annual family income, hyperlipoidemia, Hypertension was 0.95 (0.93, 0.98), *p* = 0.0004. This illustrated that chest pain incidence rose by 5% for every 1 h increase in sleep duration. The categorical variable of sleep duration processing exhibited a similar pattern (*P* < 0.0001).

**Table 2:** Relationship between sleep duration and chest pain.

**Variable**	**Model I** **(OR, 95%CI)**	**p**	**Model II** **(OR, 95%CI)**	***P***	**Model III** **(OR, 95%CI)**	***P***
Sleep duration (h)	0.96(0.94,0.99)	0.0040	0.95 (0.93,0.98)	0.0004	0.95 (0.93, 0.98)	0.0004
Duration of sleep (h)					
<6	Ref		Ref		Ref	
≥6, <8	0.59 (0.52, 0.66)	<0.0001	0.58 (0.52, 0.66)	<0.0001	0.66 (0.58, 0.75)	<0.0001
≥8	0.64 (0.57, 0.72)	<0.0001	0.62 (0.55, 0.70)	<0.0001	0.66 (0.58, 0.75)	<0.0001

*Model I, not adjusted*.

*Model II, we adjust age and gender*.

*Model III, we adjust age and gender, BMI, diabetes, race, marital status, annual family income, hyperlipoidemia, Hypertension*.

**Figure 2:**
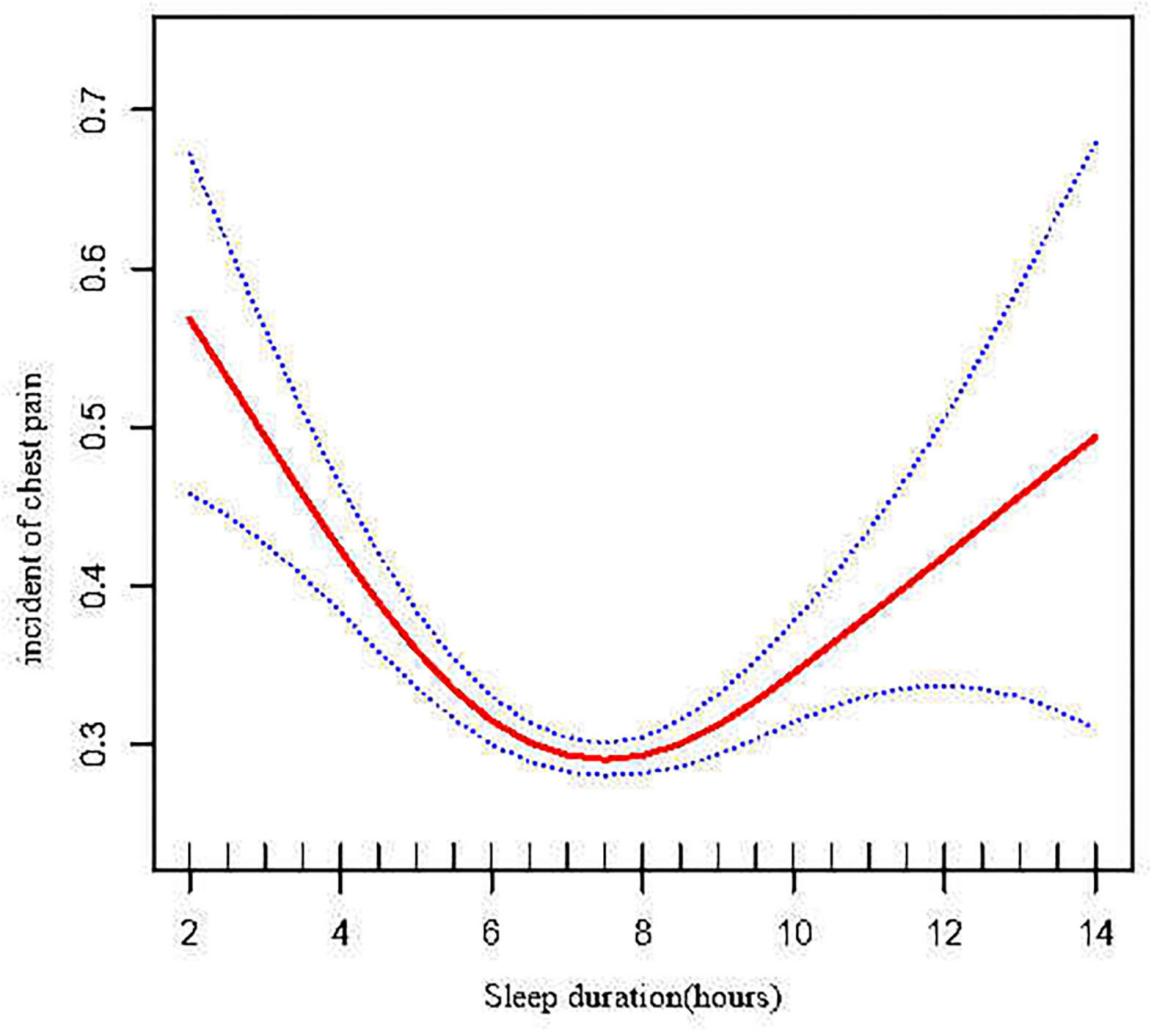
Non-linear relationship of sleep duration and chest pain.

A generalized additive model (GAM) was adopted in further assessment to explore the relationship of duration of sleep with chest pain (Figure 2), which exhibited that there was a non-linear association of duration of sleep with chest pain. Threshold effect assessment *via* piecewise linear regression (Table 3) illustrated that when sleep duration was <6.5 h, the chest pain incidence was negatively linked with sleep duration[OR = 0.77 (0.72, 0.82) P < 0.0001]. Nonetheless, when the sleep duration was ≥6.5 h, chest pain incidence rose with increasing sleep duration [OR = 1.07 (1.03, 1.11) *P* = 0.0014]. According to this analysis, 6.5 h of sleep was found to be the optimal duration of sleep relating to chest pain incidence (Figure 2).

**Table 3:** The result of two-piecewise linear regression mode.

**Inflection point of sleep duration**	**OR, 95%CI**	***P***
Sleep duration <6.5	0.77 (0.72, 0.82)	<0.0001
Sleep duration ≥6.5	1.07 (1.03, 1.11)	0.0014
*P* for log likelihood ratio test		<0.001

*Fully adjusted adjusts for age and gender, BMI, diabetes, race, marital status, annual family income, hyperlipoidemia, Hypertension*.

## Discussion

From the logistic regression analysis we found the duration of sleep was an independent factor in the occurrence of chest pain and we also found a U-shaped association of duration of sleep with incident chest pain. The optimal duration of sleep to minimize the risk of chest pain was around 6.5 h. Not only excessive sleep, but short duration of sleep would also increase chest pain incidence.

Chest pain is among the chief complaints, which is always met in clinic and generally looked into as a possible symptom of coronary artery disease (1). However, In approximately half of the cases, chest pain is of Non-cardiac origin, such as esophageal disorder (9), depression (10), psychiatric disease (11) and is frequently the result of musculoskeletal diseases (12). According to epidemiological studies, pain is linked to poor sleep quality and lack of sleep time (13), greater sleep fragmentation (14). Adults with acute pain are additionally less likely to report having a good sleep (15) and evening pain as a predictor of overnight sleep, (16). According to a national survey, pain assessments increase in a U-shaped curve as a function of previous sleep time (17) and our findings were in agreement with this result.

Short sleep, often known as sleep loss, is defined as sleeping for fewer than the required 6 h each night (5, 6) and is related with diverse metabolic, cardiovascular, as well as mental comorbidities. Sleep deprivation has a negative impact on daily wellbeing and is quite frequent among adults in the us, with over one-third reporting sleeping <6 h each night (7, 18). In some investigations (16) showed that a short duration of sleep is additionally related with an elevated next-morning pain intensity among the youth and poorer sleep in the nighttime forecasting higher next day pain. Edward‘s study (17) found that sleep and pain correlations over an 8-day period and discovered that poor sleep time (6 h) was associated with an escalation in the frequency of pain reporting in the general population.

While there is much knowledge on the negative health consequences of inadequate sleep, comparatively less emphasis is paid to the hazards linked to excessive sleep. Only few studies have assessed that long sleep duration also increased occurrence of pain (19, 20) and our findings were in agreement with those of the author. The responsible mechanisms of the association among excessive sleep and chest pain remain to be explored (21). The pain-to-sleep connection is a bi-directional one: Pain disrupts sleep, and short or disturbed sleep in turn causes pain thresholds to decrease and increases the frequency of spontaneous aches and pain. Numerous experimental, clinical, and review investigations have already examined this connection (22–24). A possible explanation is that changes in the mono-aminergic along with opioidergic cascades participate in pathophysiology of some pain conditions (25, 26). Afolalu et al. (13) illustrating that sleep issues are prospectively linked to pain outcomes and they discovered that the inflammatory profile may play a role in mediating pain responses. Immune system activation is prevalent during sleep and pain. Pro-inflammatory-cytokines consisting of IL-1, IL-6, and TNF- are recognized to play an indispensable in the progress of inflammatory and neuropathic pain (27). A meta-analysis of cohort investigations found that sleep disruption and lengthy sleep duration, but not short sleep duration, were linked to elevated levels of systemic inflammatory biomarkers (CRP and IL-6) (28).

Collectively, our study has reported the relationship of sleep duration with chest pain. Insufficient sleep and excessive sleep both increase the risk of chest pain. There are numerous limitations in our research work. Firstly, we were not able to infer causation from the data due to the cross-sectional aspect of our research. Because there appears to be a bi-directional link connecting sleep time with cheat pain. Additionally, the data from the Rose Questionnaire could be affected by “interviewer bias.” Thirdly, because our investigation only contained one program that inquired about average sleep length, we were unable to investigate the reasons behind people's short or long sleep or the various types of sleep disorders they suffer from. Distinct demographic or psychological variables, for instance poor socio-economic level or high Neuroticism, may be linked to different causes of inadequate sleep. To develop more relevant and effective sleep health therapies, additional research on these subtle aspects is required.

## Conclusion

In this study of US adults, we find that sleep duration were independently associated with the incidence of chest pain. Chest pain risk was lowest when sleep duration was around 6.5 h. Not only insufficient sleep but excessive sleep also increases the risk of chest pain.

## Data availability statement

The original contributions presented in the study are included in the article/supplementary material, further inquiries can be directed to the corresponding author/s.

## Ethics statement

The studies involving human participants were reviewed and approved by the National Center for Health Statistics ethics review board. The patients/participants provided their written informed consent to participate in this study. Written informed consent was obtained from the individual(s) for the publication of any potentially identifiable images or data included in this article.

## Author contributions

All authors listed have made a substantial, direct, and intellectual contribution to the work and approved it for publication.

## Conflict of interest

The authors declare that the research was conducted in the absence of any commercial or financial relationships that could be construed as a potential conflict of interest.

## Publisher's note

All claims expressed in this article are solely those of the authors and do not necessarily represent those of their affiliated organizations, or those of the publisher, the editors and the reviewers. Any product that may be evaluated in this article, or claim that may be made by its manufacturer, is not guaranteed or endorsed by the publisher.

## Abbreviations

BMI: Body mass index
ED: emergency department
NHANES: National Health and Nutrition Examination Survey
NCHS: National Center for Health Statistics
CAPI: computer-facilitated personal interview
GAM: generalized additive model.
